# Optic nerve changes detected with ocular ultrasonography during epiduroscopy: a narrative review

**DOI:** 10.3389/fsurg.2026.1603586

**Published:** 2026-03-17

**Authors:** İlker Çöven, Yasin Tire, Aydın Mermer, Abdullah Celep, Serhat Cömert

**Affiliations:** 1 Department of Neurosurgery, Health and Science University Konya Training and Research Hospital, Konya, Türkiye; 2 Department of Anesthesiology and Reanimation, Konya City Hospital, University of Health Sciences, Konya, Türkiye; 3 Outcomes Research Consortium, Houston, TX, United States; 4 Department of Neurosurgery, Yildirim Beyazit University Yenimahalle Training and Research Hospital, Ankara, Türkiye

**Keywords:** epiduroscopy, intracranial pressure (ICP), non-invasive monitoring, ocular ultrasonography (OUS), optic nerve sheath diameter (ONSD), spinal procedures

## Abstract

**Background:**

Epiduroscopy has emerged as a transformative minimally invasive tool for the diagnosis and treatment of spinal diseases. However, the infusion of irrigation fluids and manipulation of epidural structures during the procedure may unintentionally alter intracranial dynamics, leading to transient increases in intracranial pressure (ICP).

**Objectives:**

This narrative review aims to evaluate the relationship between epiduroscopy and changes in optic nerve morphology, advocating for the use of ocular ultrasonography (OUS) as a non-invasive monitoring tool.

**Methods:**

We consolidated contemporary literature regarding the pathophysiological foundations of optic nerve sheath diameter (ONSD) changes and the clinical utility of OUS during spinal interventions.

**Results:**

Ocular ultrasonography allows for real-time, bedside measurement of ONSD, which serves as a reliable indirect indicator of ICP. The optic nerve sheath, being continuous with the subarachnoid space, dilates in response to pressure surges transmitted from the epidural compartment during fluid irrigation. Studies have shown that dynamic monitoring of ONSD can guide intraoperative decisions, such as adjusting irrigation pressure or volume.

**Conclusion and future directions:**

While OUS is currently subject to operator dependency, it provides a valuable non-invasive adjunct for identifying potential increases in ICP during epiduroscopy. Integrating ONSD evaluations into monitoring protocols may support clinical decision-making and enhance procedural vigilance. Future large-scale prospective studies are needed to establish standardized intervention thresholds and improve long-term patient outcomes.

## Introduction

In the care of spinal diseases, epiduroscopy has emerged as a transformational tool, delivering diagnostic and therapeutic benefits with little invasiveness. Epiduroscopy includes both diagnostic and therapeutic benefits. This technique was initially created to visualise the epidural space; however, it has since evolved with enhanced instrumentation and procedural protocols, which now enable doctors to directly evaluate pathology, deliver drugs, and guide therapies (1). Notwithstanding these benefits, the manipulation of epidural structures and the infusion of irrigation fluids during the treatment may unintentionally induce alterations in intracranial dynamics (2).

A notable consequence is the temporary increase in intracranial pressure (ICP), which may manifest as changes in the morphology of the optic nerve. Due to the intimate physical connection between the optic nerve sheath and the subarachnoid space, elevations in intracranial pressure (ICP) frequently result in quantifiable enlargements of the optic nerve sheath diameter (ONSD) (3). Recent advancements in ocular ultrasonography (OUS) have allowed practitioners to document these alterations in real time. As a non-invasive, bedside method, OUS offers instantaneous feedback on optic nerve structure, facilitating the swift identification of prospective increases in intracranial pressure (4, 5).

**Figure 1:**
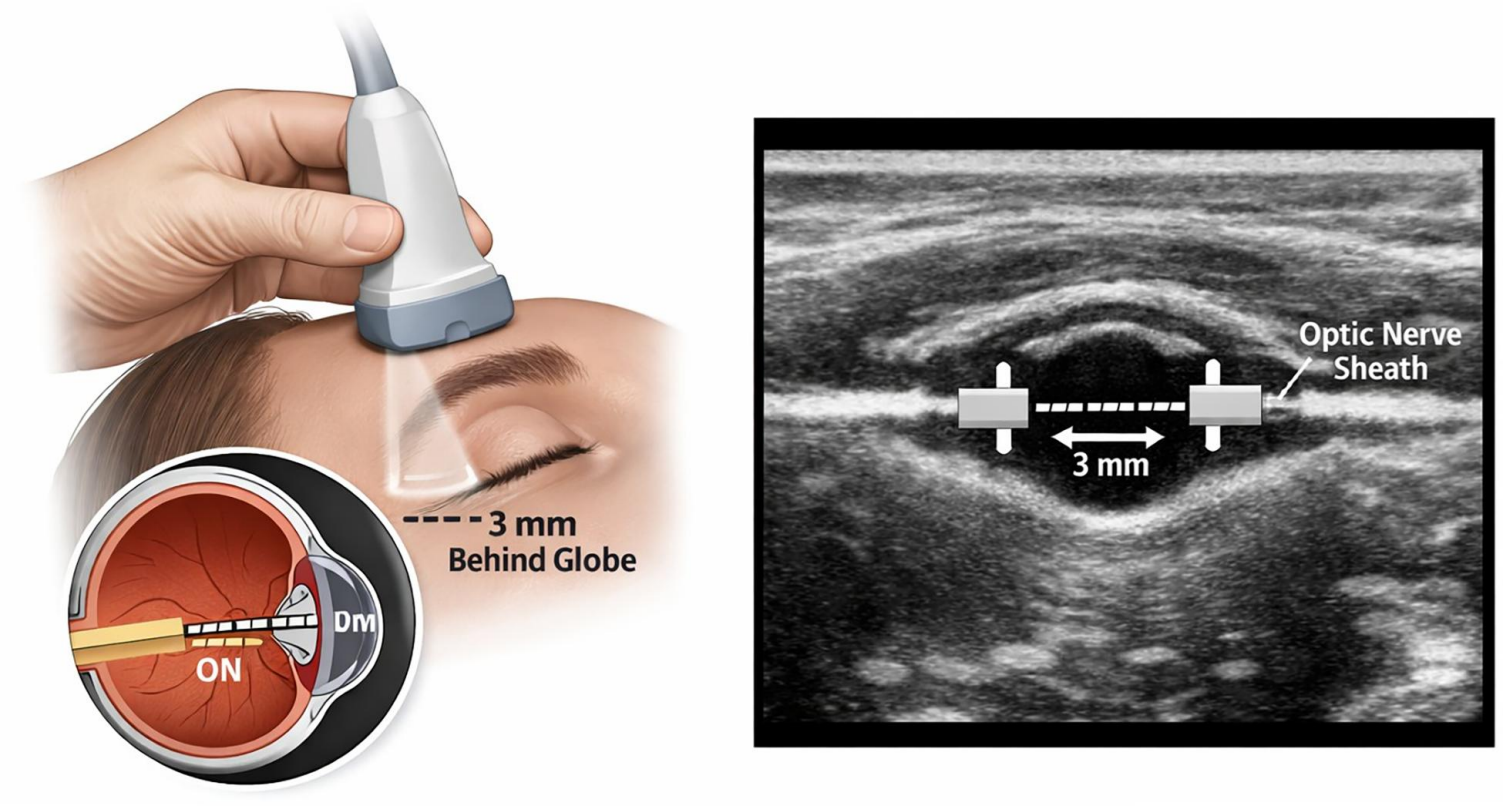
Ocular ultrasonography (OUS) and measurement of the optic nerve sheath diameter. OUS offers a non-invasive, real-time, portable, and low-cost alternative to other methods, such as CT or MRI which lacks real-time capability, or invasive ICP monitoring which is associated with procedural risks and limited practicality during epiduroscopy despite providing continuous data.

While invasive ICP monitoring is real-time but invasive, moderately expensive, and less user-friendly, CT/MRI is non-invasive but lacks real-time capabilities, is expensive, and has limited portability (6, 7). In contrast, Ocular Ultrasonography (OUS) is a non-invasive, real-time, low-cost, highly portable, and simple-to-use alternative for monitoring intracranial pressure (Table 1; Figure 1).

**Table 1:** Comparison of ocular ultrasonography (OUS) with other monitoring methods (CT/MRI and invasive ICP monitoring) based on invasiveness, real-time monitoring, cost, portability, and ease of use.

Feature	OUS	CT/MRI	Invasive ICP monitoring
Invasiveness	Non-invasive	Non-invasive	Invasive
Real-time monitoring	Yes	No	Yes
Cost	Low	High	Medium
Portability	High	Low	Low
Ease of use	Easy	Difficult	Difficult

This narrative review seeks to consolidate contemporary understanding of the relationship between epiduroscopy and alterations in the optic nerve, highlighting the efficacy of OUS as a monitoring instrument. To date, no prospective study has systematically evaluated dynamic optic nerve sheath diameter changes during epiduroscopy, and no outcome-based intervention thresholds have been established. Therefore, ocular ultrasonography should currently be regarded as a supportive decision-making tool rather than a “standalone surrogate for intracranial pressure.” By investigating the physiological foundations and therapeutic ramifications of these discoveries, we aim to advocate for more standardised monitoring techniques to improve patient safety during epiduroscopy (Table 2).

**Table 2:** Previous studies evaluating ONSD changes relevant to epiduroscopy.

Author (year)	Population	Procedure	ONSD change	Clinical implication
Tire et al., 2019 (3)	Adult pain pts	Epiduroscopy	↑ ONSD during irrigation	ICP vigilance
Beyaz et al., 2021 (10)	ENLD pts	Epiduroscopic laser	Significant ONSD ↑	Adjust irrigation
Robba et al., 2018 (6)	Mixed ICU	ICP correlation	Cut-off ∼5.5 mm	Non-invasive ICP

## Evolution and utility of epiduroscopy

In the last twenty years, epiduroscopy has become a vital method in pain management and spinal diagnostics (8). Epiduroscopy, once limited to patients with intractable spinal pain or radiculopathy, now encompasses a wider range of uses, including the assessment of epidural fibrosis and the precise administration of drugs. Its minimally invasive characteristics diminish the necessity for open surgical procedures, shorten recovery duration, and provide the combined advantages of visualisation and intervention (9).

## Pathophysiological considerations

Epiduroscopy is not risk-free, despite its advantages. The possibility of elevated ICP is a serious but little-known consequence (3). Fluid overload may occur from the administration of irrigation fluids, which are utilised to expand the epidural space for better visualisation. Furthermore, the dynamics of the distribution of cerebrospinal fluid (CSF) may change if the epidural space is mechanically altered. These circumstances have the potential to temporarily raise ICP in vulnerable patients, which is a shift that is both measurable and clinically significant (10).

The optic nerve, enclosed in a sheath that is continuous with the brain's subarachnoid space, is highly sensitive to variations in cerebrospinal fluid pressure. In instances of elevated intracranial pressure, the optic nerve sheath dilates, serving as an indirect yet dependable indicator for assessing intracranial dynamics (11). These pathophysiological insights underscore the necessity for vigilant monitoring during epiduroscopy, particularly in patients with predisposing factors for elevated ICP.

## Fundamentals of OUS

**Figure 2:**
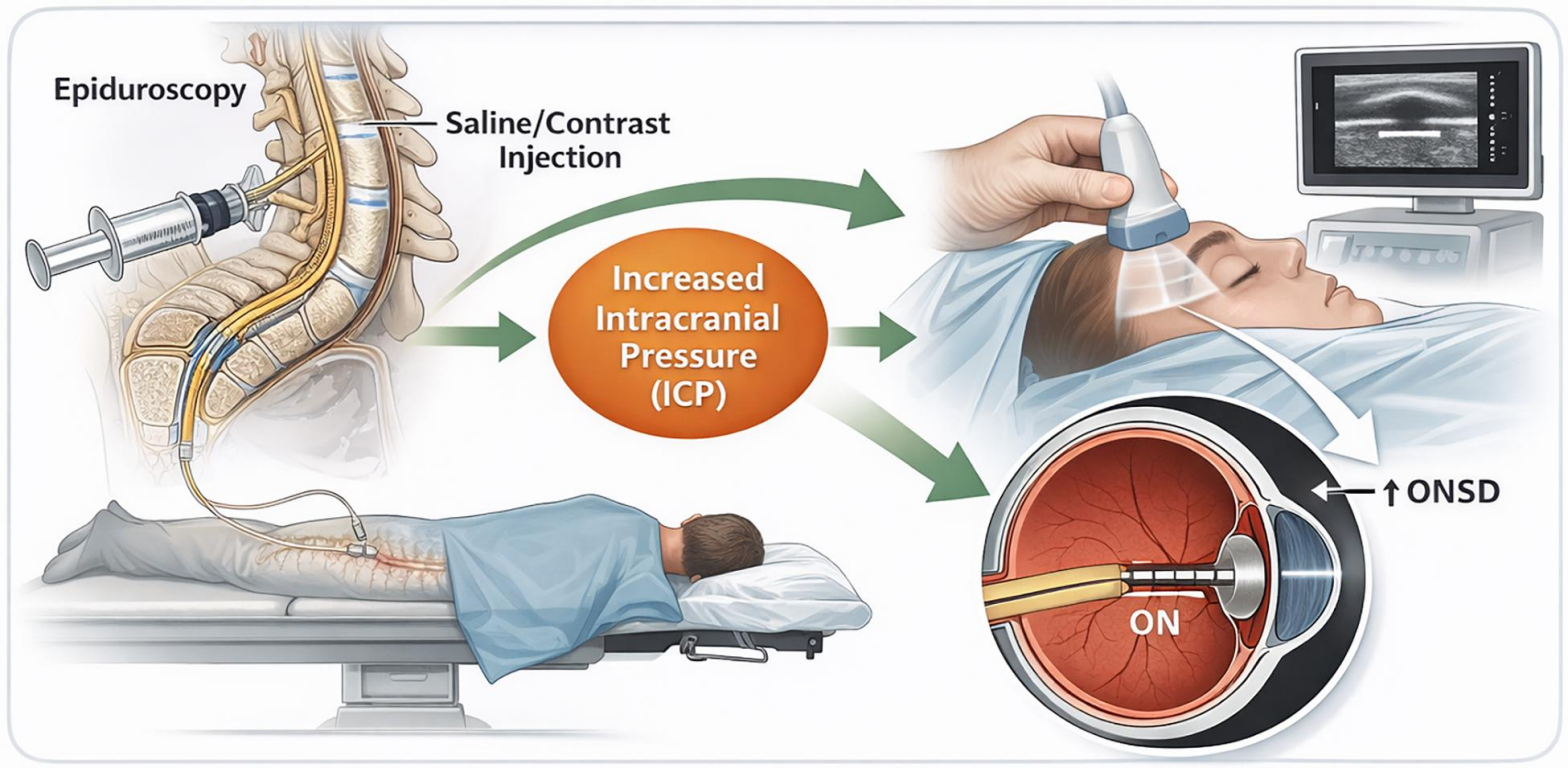
Schematic illustration of optic nerve sheath diameter (ONSD) measurement using ocular ultrasonography during epiduroscopy. Increased epidural irrigation pressure during epiduroscopy may lead to transient elevations in intracranial pressure, which are transmitted to the optic nerve sheath through the subarachnoid space. These changes can be detected as ONSD enlargement by ocular ultrasonography, typically measured 3 mm posterior to the globe. OUS enables non-invasive, real-time bedside monitoring of intracranial pressure dynamics during the procedure.

Ocular ultrasonography utilises high-frequency sound waves to produce pictures of eye structures. This approach excels in visualising the optic nerve, facilitating accurate measurements of the ONSD. The core premise is based on the transmission of ultrasonic waves that, upon contacting tissue boundaries, are reflected back to the transducer (12). Through the analysis of these echoes, physicians can recreate an image of the optic nerve and assess the surrounding sheath. A schematic illustration of optic nerve sheath diameter measurement using ocular ultrasonography and its relationship with intracranial pressure changes during epiduroscopy is provided in Figure 2.

## Intraoperative setup and practical considerations for ocular ultrasonography

Ocular ultrasonography can be safely and effectively performed intraoperatively with minimal disruption to the surgical workflow. In most reported settings, patients are positioned supine with the head maintained in a neutral position. A high-frequency linear ultrasound probe (typically 7–15 MHz) is gently placed over the closed eyelid using an adequate amount of ultrasound gel, ensuring minimal pressure is applied to avoid ocular compression. Routine sterile precautions are generally not required; however, careful attention to eye protection and operator technique is essential to ensure patient safety (12).

Optic nerve sheath diameter is commonly measured approximately 3 mm posterior to the globe in the transverse plane, where changes related to intracranial pressure are most reliably detected. In the intraoperative context, baseline ONSD measurements may be obtained prior to epidural irrigation, followed by intermittent or event-triggered reassessments during epiduroscopy, particularly during periods of increased irrigation pressure or prolonged procedural duration. Interpretation of ONSD findings should emphasize dynamic trends rather than isolated absolute thresholds, as transient increases may reflect procedural effects on cerebrospinal fluid dynamics. When integrated into perioperative monitoring, ocular ultrasonography provides a practical, repeatable, and non-invasive method for real-time assessment of intracranial pressure changes during epiduroscopy (6).

## Measuring the optic nerve sheath diameter

A crucial element of utilising OUS is its ability to quantitatively evaluate the ONSD. Multiple investigations have established a threshold, generally between 5.0 and 5.7 mm in adults (13), beyond which a substantial connection with increased ICP is observed. The swift, bedside characteristics of OUS render it an attractive option in acute care environments, when timely choices are crucial. In the realm of epiduroscopy, the capacity to consistently observe ONSD provides an early alert mechanism for detrimental intracranial occurrences (14).

## Advantages in the clinical setting

OUS is non-invasive and reproducible, making it a valuable instrument in dynamic clinical situations. In contrast to conventional imaging methods, which necessitate patient relocation and result in delayed analysis, OUS offers immediate input. This immediacy is especially crucial during epiduroscopy, since fluctuations in ICP may be ephemeral yet clinically significant. Moreover, OUS is less resource-demanding than magnetic resonance imaging (MRI) or computed tomography (CT), hence reinforcing its incorporation into standard monitoring programs (15).

## Advantages and limitations of ocular ultrasonography during epiduroscopy

### Advantages

- Non-invasive and repeatable
- Real-time, bedside monitoring
- Feasible during prone positioning
- Allows immediate procedural modification

### Limitations

- Operator dependency
- Inter-individual variability
- Lack of epiduroscopy-specific ONSD thresholds
- Indirect estimation of ICP

## Optic nerve changes during epiduroscopy

### Procedural influences on ICP

The proper application of irrigation fluids is essential for ensuring excellent visualisation of the epidural area during epiduroscopy. This essential intervention may result in fluid buildup, thereby increasing pressure inside the restricted epidural compartment. The resultant elevation in pressure can be conveyed through the dura mater and into the cerebrospinal fluid, resulting in a temporary yet quantifiable increase in intracranial pressure (16).

This mechanism can be understood mechanistically by examining the balance of craniospinal pressure. The injection of extra fluid disrupts the usual homeostatic equilibrium, activating compensatory mechanisms that may prove inadequate to avert an increase in intracranial pressure (ICP). In patients with diminished intracranial compliance such as individuals with a history of head trauma, hydrocephalus, or other intracranial disorders this pressure surge can be more significant (17).

### Early detection and intervention

Early detection of elevated intracranial pressure during epiduroscopy holds considerable clinical importance. An increased ONSD, identified by OUS, can act as a proxy indicator for intracranial hypertension, facilitating the timely initiation of remedial actions (18, 19). Interventions may involve altering irrigation fluid pressure, adjusting fluid volume, or providing drugs, such as diuretics, to alleviate fluid excess. The capacity to respond promptly to these alterations can prevent a series of consequences that could otherwise result in visual impairment, neurological abnormalities, or more serious systemic disruptions.

### Enhancing safety protocols

Incorporating OUS within the procedural workflow of epiduroscopy can significantly enhance patient safety standards. Integrating regular ONSD evaluations into the monitoring protocol enables doctors to create a continuous feedback mechanism that guides intraoperative decision-making. This integration not only improves the immediate safety of the surgery but also enhances long-term outcomes by decreasing the occurrence of problems associated with temporary ICP rises.

Moreover, the portability and user-friendliness of OUS facilitate its smooth integration into many clinical scenarios, ranging from well-equipped operating rooms to more challenging settings. The prospective uses of OUS extend beyond epiduroscopy, providing utility in several settings necessitating meticulous monitoring of intracranial dynamics.

Several observational studies and procedural reports have described real-time adjustments in epiduroscopy technique following the detection of optic nerve sheath diameter enlargement by ocular ultrasonography (3, 10). Reported interventions include reduction of epidural irrigation pressure or volume, temporary interruption of the procedure, and increased neurological vigilance when dynamic ONSD increases were observed. In some cases, these findings prompted closer postoperative monitoring or modification of procedural duration, particularly in patients with limited intracranial compliance. Although these reports are largely observational and no randomized trials have yet established outcome-based thresholds for intervention, they highlight the potential role of ocular ultrasonography as a practical decision-support tool rather than a standalone diagnostic modality during epiduroscopy (10).

### Multimodal monitoring and future innovations

Although OUS offers significant insights, its incorporation should be viewed as supplementary to other monitoring methods. Integrating OUS with neurophysiological monitoring, invasive ICP measurements, or advanced imaging techniques may provide a more thorough understanding of intracranial dynamics during epiduroscopy. Future advancements may encompass the creation of automated ONSD measurement systems that reduce operator variability and enhance the efficiency of the monitoring process. These developments are expected to enhance the accuracy and dependability of OUS, hence promoting a safer procedural environment.

## Limitations and future directions

### Operator dependency and measurement variability

Notwithstanding its benefits, the implementation of OUS presents certain problems. A primary disadvantage is its operator dependency; the precision of ONSD measurements might fluctuate considerably depending on the examiner's experience and expertise. This unpredictability requires stringent training and the implementation of standardised assessment techniques to guarantee consistency across various clinical environments. Moreover, variables including patient anatomy, location, and the specific ultrasound instrument employed can affect measurement results (20).

### Need for large-scale, prospective studies

Although existing evidence endorsing the efficacy of OUS in identifying optic nerve alterations during epiduroscopy is encouraging, it primarily originates from limited research and individual case reports. To corroborate these findings and formulate comprehensive clinical guidelines, there is an immediate necessity for extensive, prospective study. These investigations should seek to clarify the exact correlation between ONSD variations and clinical outcomes, thus establishing actionable intervention thresholds. Moreover, randomised controlled trials contrasting OUS-guided therapy with routine care would yield essential insights into the technique's efficacy and cost-effectiveness.

### Technological advances and integration

Subsequent study should concentrate on technology advancements that can alleviate the constraints of existing OUS approaches. The advancement of automated or semi-automated systems for ONSD measurement presents considerable potential, since these systems may diminish inter-operator variability and improve measurement precision (14, 21). Simultaneously, the integration of OUS data with additional physiological monitoring devices could provide a more comprehensive approach to patient management during epiduroscopy. The primary objective is to establish a multimodal monitoring framework that enhances safety and improves patient outcomes (22, 23).

## Conclusion

The utilisation of ocular ultrasonography in epiduroscopy signifies a notable progression in procedural oversight. OUS facilitates the real-time identification of alterations in the optic nerve sheath, acting as a significant surrogate marker for variations in intracranial pressure, a crucial factor in maintaining patient safety during minimally invasive spinal procedures.

The increasing popularity and scope of epiduroscopy may transform the integration of OUS into standard monitoring regimens, enhancing doctors' ability to foresee and address issues linked to temporary ICP rises. Despite the limitations of measurement variability and the necessity for comprehensive clinical validation, the advantages of OUS are evident. Further research, especially through extensive prospective studies and technical advancements, may soon establish ocular ultrasonography as a fundamental component of intraoperative monitoring in epiduroscopy, therefore improving patient safety and procedural results.

This review emphasises the significance of acknowledging the correlation between epidural fluid dynamics and optic nerve morphology. It underscores the pivotal role of OUS in reconciling procedural innovation with patient safety, a synergy that promises to enhance both clinical practice and patient outcomes in the treatment of spinal diseases.
